# Shaking Table Test of U-Shaped Walls Made of Fiber-Reinforced Foamed Concrete

**DOI:** 10.3390/ma13112534

**Published:** 2020-06-03

**Authors:** Emmanuel A. Flores-Johnson, Brenda A. Company-Rodríguez, J. Francisco Koh-Dzul, Jose G. Carrillo

**Affiliations:** 1 CONACYT-Unidad de Materiales, Centro de Investigación Científica de Yucatán, Calle 43, No. 130 Col. Chuburná de Hidalgo, Mérida, Yucatán 97205, Mexico; 2 Unidad de Materiales, Centro de Investigación Científica de Yucatán, Calle 43, No. 130 Col. Chuburná de Hidalgo, Mérida, Yucatán 97205, Mexico; brenda.company@cicy.mx (B.A.C.-R.); juan.koh@cicy.mx (J.F.K.-D.); jgcb@cicy.mx (J.G.C.); 3 Tecnológico Nacional de México/Instituto Tecnológico de Mérida, Av. Tecnológico km 4.5, Mérida, Yucatán 97118, Mexico

**Keywords:** foamed concrete, henequen fiber, natural fiber, U-shaped wall, shaking table test, dynamic cyclic loading, seismic performance, finite-element simulation

## Abstract

Fiber-reinforced foamed concrete (FRFC) is a lightweight material that has the potential to perform well in seismic applications due to its low density and improved mechanical properties. However, studies focused on the seismic assessment of this material are limited. In this work, U-shaped wall specimens, made of FRFC reinforced with henequen fibers and plain foamed concrete (PFC) with a density of 900 kg/m^3^, were subjected to shaking table tests. PFC and FRFC were characterized using compression and tension tests. FRFC exhibited enhanced mechanical properties, which were attributed to the fibers. The dynamic tests showed that U-shaped walls made of FRFC performed better than those made of PFC. The time period prior to the collapse of the FRFC U-shaped walls was longer than that of the PFC specimens, which was attributed to the enhanced specimen integrity by the fibers. Finite element simulations of the shaking table test allowed for the prediction of the stress concentration and plastic strain that may lead to the failure of the U-shaped wall. These results showed that U-shaped walls made of FRFC have the potential to perform well in seismic applications, however, these results are preliminary and further studies are needed to support the findings of this work.

## 1. Introduction

Unreinforced masonry (URM) structures, such as those made of adobe, clay, bricks, or blocks, are used in many parts of the world because they are inexpensive, relatively easy to build and utilize locally available materials. However, in many cases, URM structures are unregulated, and are built using simple technology and low-quality construction practices [1]. One major concern is that URM construction is still widely used in areas prone to seismic activity. URM structures are highly susceptible to damage from earthquakes [2], meaning that a major earthquake could cause significant human and economic losses. URM structures are particularly vulnerable to seismic events because they are not capable of dissipating energy through large plastic deformations during an earthquake, due to their lack of ductility [3]. Moreover, URM buildings may also have insufficient strength to resist seismic load due to the absence of connections between wall leaves, poor adhesion of the mortar and the presence of voids [1]. The failure of earthen buildings during an earthquake is mainly due to its low strength and brittleness [4]. For instance, the 2017 Puebla earthquake that struck the south of Mexico on 19 September, caused significant damage in the states of Oaxaca and Chiapas, where 110,000 houses were destroyed or severely damaged [5]. In these regions, a large number of URM structures are made of adobe bricks [6]. These types of structures, typically made of walls without any reinforcement and with heavy roofs made of wooden logs and clay tiles, usually exhibit the most significant damage during a seismic event [7]. The 1999 Oaxaca earthquake affected over 41,000 houses and typical damage observed in URM structures was failure of walls, including out-of-plane failure and inclined cracking [7]. During this same earthquake, plain masonry structures also performed poorly due to a lack of continuity and confinement [7]. Several studies have been carried out to understand the seismic performance of URM and cement-based structures using dynamic tests. These investigations include cyclic loading of walls and structures using actuators [8,9] and shaking table testing [10,11]. In many cases, researchers have utilized scaled models for the dynamic testing of masonry structures [12,13] since the testing of full-scale models could be costly or infeasible. Numerical simulations have also been employed to understand the dynamic loading of masonry and cement-based structures [14,15,16,17,18]. Numerical modelling is a useful tool to complement seismic experimental studies, which require expensive equipment and instrumentation [19].

Several techniques have been developed to reinforce adobe and URM structures, including confinement of walls [20], retrofitting using fiber-reinforced polymer (FRP) composites [21,22] and steel rebars [8], repair using FRP composites [23], mortar strips [24], among others. However, many of these techniques are not commonly used in poor and remotes areas. The use of lightweight construction materials to reconstruct or replace certain features of URM houses in regions prone to seismic activity, could be an alternative option for improving seismic performance of these structures. Seismic actions induce inertia forces that are proportional to the building mass and therefore lower weights in the structures will provide an enhanced seismic performance of the building under the same structural conditions [25]. Dominguez-Santos et al. [25] studied the seismic performance of frame structures built with blocks made of concrete and wood additives with a density of ~2250 kg/m^3^. They found that the frames built with the reduced-density wood-concrete blocks exhibited a superior seismic-resistant behavior when compared to those built with ordinary concrete blocks. Tomaževič and Gams [26,27] studied the seismic response of autoclaved aerated concrete (AAC) confined masonry buildings using shaking table tests. The lightweight AAC blocks had a density of ~500 kg/m^3^. They found that the prototype buildings exhibited adequate seismic behavior when constructed as confined masonry systems. Dunn et al. [28] studied the seismic performance of a reinforced lightweight foamed concrete walling. They found that their proposed precast walling and connection system would likely provide adequate resistance for low-rise residential buildings in regions of low to moderate seismicity.

Foamed concrete is a cementitious material, in which air-voids are trapped in the mortar. Foamed concrete is classified as a lightweight concrete, and its density can vary from 400 to 1600 kg/m^3^ [29]. Foamed concrete could be used as a lightweight construction material due to its low density, to effectively reduce the risk of earthquake damage [30]. However, foamed concrete is not good in supporting tensile loads, as it often cracks in the plastic state and during drying shrinkage. The flexural and tensile strengths of foamed concrete range between 15% and 35% of its compressive strength [31]. It has been reported that the inclusion of synthetic or natural fibers in foamed concrete can improve flexural and tensile strengths, and ductility [32,33,34], by changing the typical brittle behavior to elastic-plastic behavior [35]. This enhanced toughness and ductility of cement-based and earthen materials, produced by the inclusion of fibers, is very important in order to avoid sudden and catastrophic failure [35,36]. Although the inclusion of fibers in the cementitious matrix could affect the air-void size and its distribution [37,38], which in turn could affect the mechanical properties of the fiber-reinforced material [39,40], the use of low fiber volume fractions and adequate fiber dispersion in the matrix will normally result in good workability and an increase of the mechanical properties [34,37]. The interest in the use of natural fibers to reinforce cement-based materials is growing because it is possible to obtain materials with low density and good mechanical properties that promote sustainability [41]. In addition, natural fibers are low-cost renewable materials that can be found in abundant supply in many countries [42]. Recent investigations performed on foamed concrete reinforced with natural fibers, such as kenaf [33], coir [43], sisal [44], and henequen [45] fibers, among others, have found that there is an enhancement of the compressive, tensile, and flexural strengths when compared to non-reinforced foamed concrete.

Based on the aforementioned literature, fiber-reinforced foamed concrete (FRFC) has the potential to perform well in structural and seismic applications due to its low density and improved toughness and ductility by the fibers, effectively reducing the risk of earthquake damage, however, studies focusing on the seismic assessment of this type of material are limited in the literature. Hence, the objective of the present study is to investigate the seismic performance of FRFC with a density of 900 kg/m^3^, reinforced with the natural fiber henequen (*Agave fourcroydes* Lem.), which is one of the main cultivated species of *Agave* used to produce fibers in the south of Mexico. For this investigation, the dynamic cyclic loading response of U-shaped wall model specimens made of FRFC was studied by means of shaking table tests with a sinusoidal excitation, and the results were compared with those of non-reinforced foam concrete specimens. The mechanical characterization of the foamed concrete was performed using compression and tension tests. Finite-element (FE) simulations of the dynamic loading of U-shaped walls were performed to predict stress concentration that may lead to failure and improve the design of the U-shaped walls.

## 2. Materials and Methods

### 2.1. Fiber-Reinforced Foam Concrete

**Figure 1:**
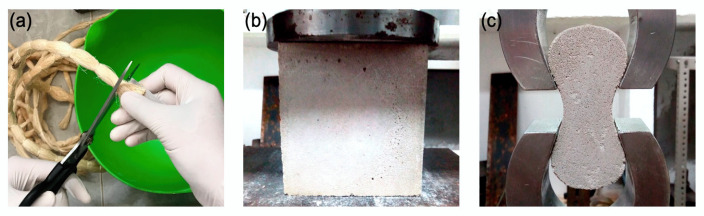
(**a**) Henequen fibers; (**b**) compression test set-up; and (**c**) tension test set-up.

The foamed concrete mixture used in this study to fabricate the U-shaped wall specimens was prepared using the pre-foaming method [35], which consisted of 3 stages: (1) preparation of the mortar, (2) preparation of the foam, and (3) mixing of mortar and foam. A target dry density of 900 kg/m^3^ was used. The mixture was prepared using the following constituents for 1 m^3^: Portland cement CPC 30R EXTRA (CEMEX, Merida, Mexico) with a minimum strength of 30 MPa at 28 days (427 kg), dry sand (214 kg), water (299 kg), and foam (EABASSOC foaming agent, EAB Associates, Altrincham, UK) (24 kg). The materials were mixed in a 60-L mixer STARK (Stark Tools USA, Chino, CA, USA) until a homogeneous mixture was obtained. The mixture without fibers is referred to as plain foamed concrete (PFC). For the fiber-reinforced foamed concrete (FRFC), henequen fibers were gradually distributed, at random, by hand in small amounts into the mixture at the end of stage 3, at a fiber volume fraction of 0.5%. Henequen fibers were supplied by local producers (Desfibradora La Lupita, Baca, Mexico). A fiber length of ~4 mm was used (Figure 1a). As a reference, the reported mechanical properties of henequen fibers are: density of 1400 kg/m^3^, elastic modulus of 13.2 GPa, tensile strength of 500 MPa, and diameter of 170 µm [46]. Since the main purpose of this work was to evaluate the effect of the presence of fibers on the dynamic response of the U-shaped walls made of FRFC, fiber treatment to improve durability was not performed.

### 2.2. Mechanical Properties of Foamed Concrete

To evaluate the mechanical behavior of the foamed concrete mixtures, uniaxial compression tests were performed according to the BS-EN-12390 using cubical specimens with dimensions of 100 mm × 100 mm × 100 mm (Figure 1b). The specimens were tested in a Shimadzu AG-1 universal testing machine (Shimadzu Corporation, Kyoto, Japan), equipped with a load cell of 100 kN, at a crosshead speed of 2.4 mm/min. The tensile behavior of the foamed concrete mixtures was also evaluated in accordance with the ASTM C307 (ASTM International, West Conshohocken, PA, USA) using dog-bone shaped specimens with a length of 76 mm and a cross-section area of 25.4 mm × 25.4 mm. The specimens were placed in a Shimadzu AG-1 universal testing machine, equipped with a load cell of 20 kN, and loaded in direct uniaxial tension at a displacement rate of 1 mm/min until failure (Figure 1c). All specimens were left to cure for at least 28 days before testing.

**Figure 2:**
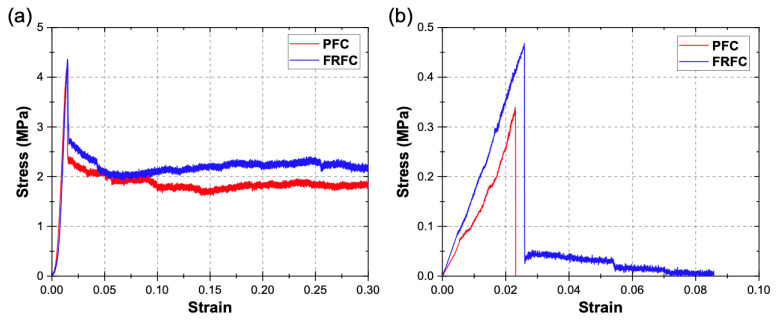
(**a**) Typical uniaxial compression stress-strain curves for plain foamed concrete (PFC) and fiber-reinforced foamed concrete (FRFC) and (**b**) typical uniaxial tension stress-strain curves for PFC and FRFC.

Figure 2a shows typical stress-strain curves for the PFC and FRFC specimens obtained from the compression tests. It can be seen in Figure 2a that the foamed concrete specimens exhibit an initial elastic response at very low strains, until a peak strength is reached at a strain of ~0.014, followed by a sudden drop in stress, which corresponds to the initial failure of the specimen. After the elastic regime, both PFC and FRFC specimens can withstand a percentage of the maximum strength at increasing strains (39% and 50% of the maximum strength for PFC and FRFC specimens, respectively, at a strain of 0.15). This percentage is higher for the FRFC, which is attributed to the enhanced integrity and toughness of the specimens by the fibers. Figure 2b shows typical stress-strain curves from the uniaxial tension tests for both PFC and FRFC. For PFC, an elastic regime is observed until the peak strength is reached at a strain of 0.023, which is followed by a sudden failure of the specimen. For FRFC, the fibers prevented a sudden failure of the specimen; after the peak strength is reached at strain of 0.027, specimens can withstand a percentage of the maximum strength as the displacement increases (9.2% of the maximum strength at a strain of 0.05). Moreover, an increase of 37% in the tensile strength was observed for the FRFC specimens when compared to the PFC.

Table 1 shows the mechanical properties of the foamed concrete mixtures, obtained from the compression and tension tests, which include the compressive and tensile strengths. The results are presented as mean ± standard deviation of at least three repetitions. It can be seen that, in general, the fiber-reinforcement enhanced the mechanical properties of the FRFC when compared to those of the PFC, which is attributed to the enhanced specimen integrity produced by the fibers [35]. It is noted that compressive elastic strain was not accurately measured due to the lack of strain gauges. For a foamed concrete with a density of 900 kg/m^3^, the elastic modulus is in the range of 1.5–2 GPa [47,48]. The density *ρ* of the mixtures shown in Table 1 was calculated using *ρ* = *W*/*V*, where *W* (kg) is the weight of the cubical specimens, measured using an analytical balance Ohaus EB6 (Ohaus Corporation, Parsippany, NJ, USA), and *V* (m^3^) is their volume. *V* was calculated using the dimensions measured by a digital caliper with a resolution of 0.05 mm (Truper, Mexico City, Mexico).

### 2.3. U-Shaped Wall Specimens

**Figure 3:**
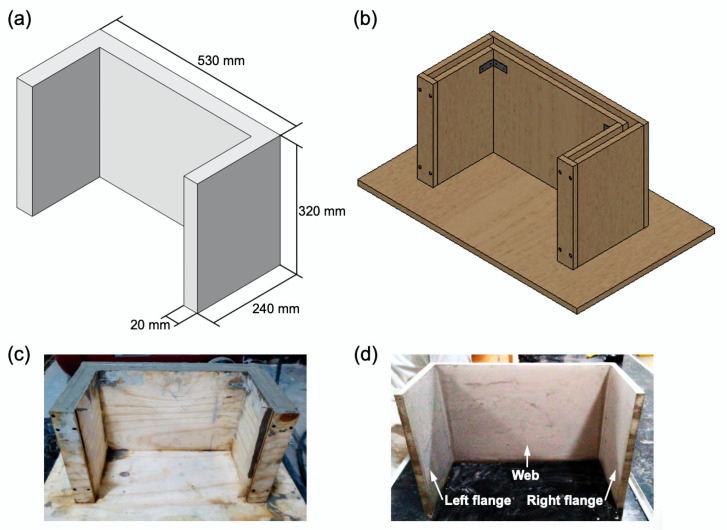
(**a**) Schematic of the U-shaped wall showing the geometrical details; (**b**) schematic of the wooden mold; (**c**) foamed concrete casting; and (**d**) U-shaped wall specimen.

To investigate the dynamic cyclic behavior of foamed concrete walls, 1:7.5 scaled U-shaped wall models were fabricated. The scaled U-shaped wall structure is intended to represent a wall section of a typical house, in the rural areas, in the state of Oaxaca, Mexico [49,50]. The geometrical details of the U-shaped wall with a cross-section thickness of 20 mm are described in Figure 3a. Although a preliminary study showed that adequate fiber dispersion can be achieved in the mixtures for fiber volume fractions of up 1%, a fiber volume fraction of 0.5% was selected for this work to ensure adequate fiber dispersion and workability of the mixture, considering the thickness of the U-shaped walls. However, further studies should be performed using different fiber volume fractions to find a balance between workability and mechanical properties. To fabricate the U-shaped walls, a mold was manufactured as shown in Figure 3b. The foamed concrete mixture was prepared as aforementioned and cast into the mold (Figure 3c). The U-shaped wall specimens were left to cure for at least 28 days at room temperature before testing. In total, six U-shaped wall specimens were fabricated, i.e., three specimens using the PFC mixture and three specimens using the FRFC mixture (Figure 3d). The average mass of the U-shaped wall specimens made of PFC and FRFC was 5.09 and 5.44 kg, respectively.

### 2.4. Shaking Table Test and Instrumentation

**Figure 4:**
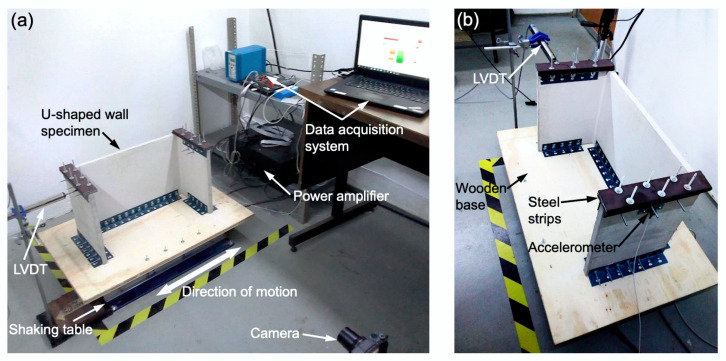
(**a**) Shaking table test set-up and (**b**) linear variable differential transformer (LVDT) and accelerometer positions.

The U-shaped wall specimens were subjected to dynamic cyclic loading using a single-axis shaking table Quanser Shake Table II (Quanser Consulting Inc., Markham, ON, Canada) [51]. Figure 4a shows the test set-up, in which, the U-shaped wall specimen is mounted on a wooden base, with dimensions of 800 mm × 500 mm × 18 mm, using steel slotted angles. The wooden base is then attached to the shaking table using screws and nuts. A mass of 1.4 kg was mounted at the top of each flange using steel strips (Figure 4b), to simulate the mass of a roof [52]. Slotted angles were used to connect the steel strips to the flanges. The average total mass of the structure (base, wall, steel strips and connectors) was 13.25 kg. The U-shaped wall specimens were subjected to a 4-Hz sinusoidal base motion with an amplitude of 20 mm, parallel to the web segment (Figure 3d and Figure 4a), until failure occurred. A linear variable differential transformer (LVDT) Micro Measurements HS100 (Vishay Precision Group, Malvern, PA, USA), with a displacement range of 100 mm, was used to record the displacement of the left flange (Figure 4b). A single-axis accelerometer Kistler 10 g PiezoBeam (Kistler Group, Winterthur, Switzerland), with a range of ±98 m/s^2^, was attached to the right flange to record its acceleration history during the test (Figure 4b). A fast camera Sony Cyber-shot DSC-RX100 IV (Sony Corporation, Tokyo, Japan) recording at 120 frames per second was used to capture the failure of the U-shaped wall specimens during the shaking table test (Figure 4a).

**Figure 5:**
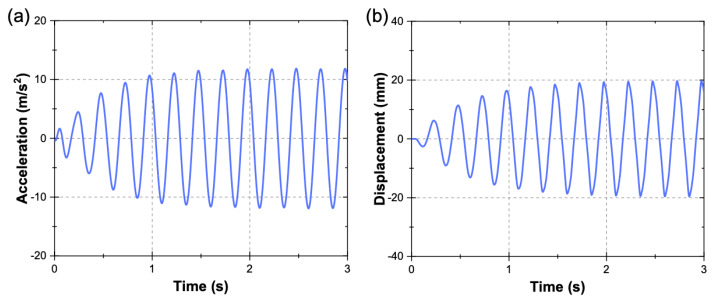
Acceleration and displacement measured by the built-in sensors of the shaking table: (**a**) acceleration–time history and (**b**) displacement–time history.

Figure 5a,b show the acceleration–time and displacement–time histories, respectively, measured by the built-in sensors of the shaking table. A 10-Hz low-pass digital filter was applied to the acceleration signal in order to eliminate noise. It can be seen in Figure 5 that after ~1.5 s, the maximum acceleration and displacement of the shaking table are around ±12.5 m/s^2^ and ±19.8 mm, as expected, for the abovementioned sinusoidal excitation parameters.

### 2.5. Numerical Simulations

**Figure 6:**
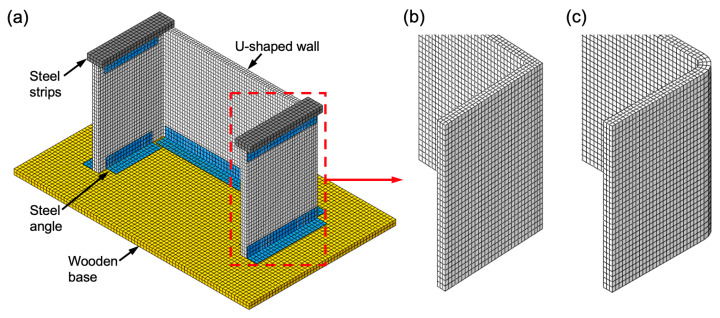
(**a**) Finite-element (FE) mesh of 3D model of U-shaped wall subjected to shaking table testing; (**b**) U-shaped wall with flat corners; and (**c**) U-shaped wall with round corners.

The numerical simulations of the U-shaped wall, subjected to dynamic cyclic loading, were performed using the finite-element (FE) software Abaqus/Explicit (Version 2016, Dassault Systèmes Simulia Corp., Providence, RI, USA) [53]. The FE simulation was employed as a tool to investigate localized stress concentration and plastic strain that may lead to cracking and failure during the dynamic loading. The geometry of the 3D FE model was based on the U-shaped wall and shaking table test described in Section 2.3 and Section 2.4, respectively. The FE model consisted of a U-shaped wall, wooden base, steel angles, and steel strips (Figure 6a). The mesh comprised 8-node brick elements (C3D8R) with reduced integration formulation. The base, steel strips and angles were modelled as rigid bodies. A global element size of 10 mm was used. For the U-shaped wall, an average element size of 7 mm was used, giving a total of 11,520 elements. The steel angles were tied to the base, steel strips and wall using the Tie Constraint option (fully bonded).

Steel parts and wooden base were modelled using densities of 7800 and 500 kg/m^3^, respectively. The elastic behavior of the foamed concrete material used for the U-shaped wall was modelled using a compression elastic modulus of *E* = 2 GPa, Poisson’s ratio of *ν* = 0.05 and density of *ρ* = 900 kg/m^3^. The plastic behavior was modelled using the Crushable Foam material model with the following parameters: compression yield stress ratio *k* = 1 and hydrostatic yield stress ratio *k_t_* = 0.1 [35]. For the Crushable Foam model hardening tabular data, experimental curves from the uniaxial quasi-static compression test (Figure 2a) were used [54]. A failure criterion, to simulate failure of the material, was not implemented due to the lack of fracture parameters.

Gravity load was applied to the entire model as a vertical acceleration field (−9.806 m/s^2^). To simulate the dynamic cyclic loading of the shaking table, the displacement–time history curve from Figure 5b was input in tabular form and applied to the wooden base as a prescribed displacement boundary condition. The total time of the simulated dynamic loading was 3 s. To investigate the effect of the shape of the wall corners on the stress concentration, in addition to the wall with flat corners (Figure 6b), a wall with round corners (inner and outer radii of 20 and 40 mm, respectively) was also modelled (Figure 6c).

**Figure 7:**
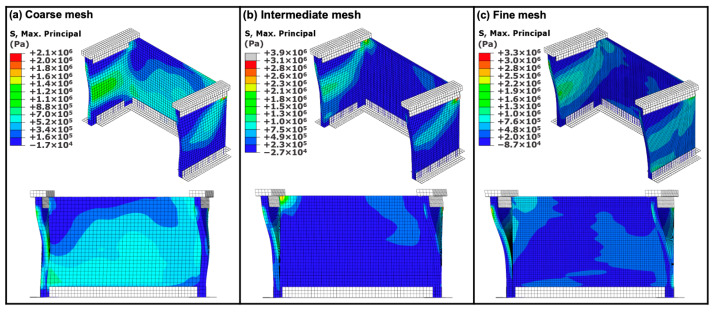
Contour plots of the maximum principal stress for the U-shaped wall subjected to dynamic loading (*t* = 1.89 s) using different element sizes: (**a**) 10 mm (course mesh); (**b**) 7 mm (intermediate mesh); and (**c**) 5 mm (fine mesh); (for illustration purposes, the deformation in the direction of motion has been scaled up by a factor of 50 to visualize the bending of the specimens).

A mesh sensitivity analysis was carried out using three different element sizes: 10 mm (coarse mesh), 7 mm (intermediate mesh) and 5 mm (fine mesh), resulting in 2, 3, and 4 through-thickness elements, and a total of 4374, 11,520, and 34,556 elements, respectively. Figure 7 shows the contour plots of the predicted maximum principal stress for the U-shaped wall with flat corners subjected to dynamic loading (*t* = 1.89 s) using different element sizes. It is noted that in Figure 7, for illustration purposes, the deformation in the direction of motion has been scaled up by a factor of 50 to visualize the bending of the specimens. It can be seen that the stress contours are similar for the intermediate (Figure 7b) and fine (Figure 7c) meshes, while for the course mesh, the stress contour is different (Figure 7a). The model using the intermediate mesh predicted a maximum stress value ~18% higher than that of the model using the fine mesh, while the model using the course mesh predicted a maximum stress value ~36% lower than that of the model using the fine mesh. The fine mesh could improve the accuracy of the solution; however, this results in a significant increase in the computational cost. Based on this analysis, the intermediate mesh size was deemed sufficient for convergence and used throughout this work to increase the computational efficiency.

## 3. Results and Discussion

### 3.1. Experimental Results

**Figure 8:**
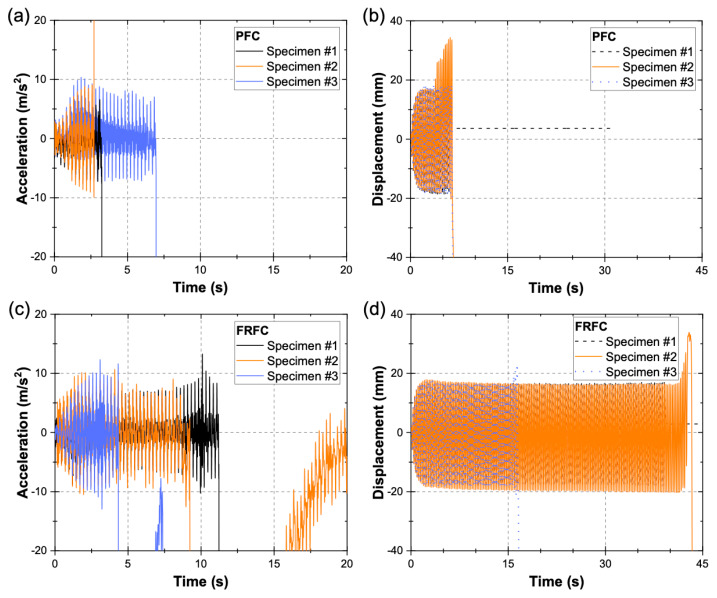
Time-history plots of (**a**) acceleration and (**b**) displacement for PFC specimens. Time-history plots of (**c**) acceleration and (**d**) displacement for FRFC specimens.

Figure 8a,b shows the acceleration–time and displacement–time histories recorded by the accelerometer and LVDT, respectively, for the PFC specimens, while Figure 8c,d show similar data for the FRFC specimens. It can be seen in Figure 8a,c that there are large acceleration values (>20 m/s^2^), which indicate the collapse of the right flange. In Figure 8b,d, displacements larger than 20 mm correspond to the collapse of the left flange. For the PFC Specimen #1, the test stopped prematurely, after the collapse of the right flange. It is noted that for some specimens, the right flange (RF) was the first to fail, while for other specimens, the left flange (LF) was the first to fail. It is also noted that for some specimens both flanges collapsed during the test, while for other specimens, only one flange collapsed. Table 2 shows the period of time from the beginning of the test to the formation of the first visible crack, and then to the time of collapse of the first flange. In the cases where a second flange failed, Table 2 also shows the period of time from the beginning of the test to the formation of the first visible crack on the second flange, and then to the time of collapse of the second flange. The time periods were calculated based on the video recording of each test. The data presented in Table 2 show that the FRFC specimens performed better than the PFC specimens during the dynamic tests. This can be seen by comparing the time periods prior to the collapse of the first flange, which in most cases is longer for the FRFC specimens when compared to the PFC specimens (on average 16.2 s longer). It is also noted that the time period between the formation of the first visible crack on the first flange and the collapse of that same flange was longer in all cases for the FRFC specimens when compared to the PFC specimens (on average 2.3 s longer). This is attributed to the fiber-reinforcement, which enables a distributed growth of micro-cracks in the specimen [55] prior to the development of macro-cracks, however, microscopic studies should be performed to confirm this assumption. It can also be seen in Table 2 that most PFC specimens exhibited failure of the second flange, while for the FRFC specimens, this only occurred in the FRFC specimen #3 after more than 16 s. This confirms the enhanced integrity of the FRFC specimens by the fibers when compared to the PFC specimens. It is important to note that there were large variations in the time of collapse of the first flange for the FRFC specimens when compared to the PFC specimens. It is believed that although every precaution was taken to achieve adequate fiber dispersion in the matrix, it is possible that fiber bundles and large air-voids could have formed in some parts of the specimens, which in turn, may have led to the initial cracking, however, microscopic studies should be conducted to verify this supposition.

**Figure 9:**
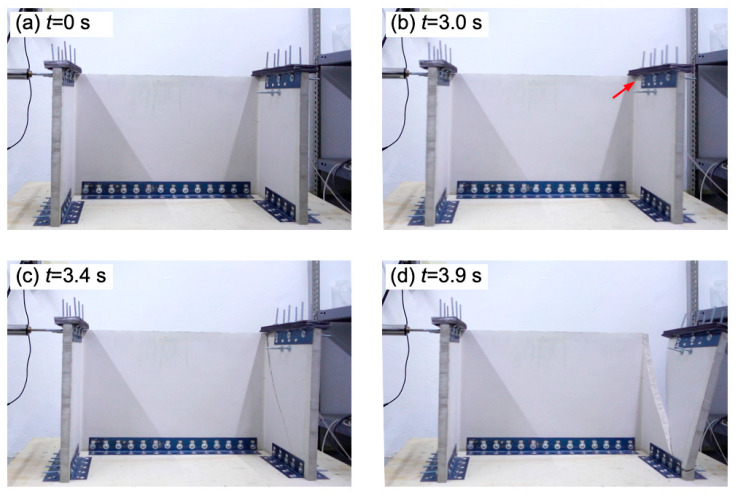
Collapse sequence of PFC specimen #1: (**a**) start of the test; (**b**) formation of the first visible crack on the right flange (indicated by a red arrow); (**c**) development of the crack; (**d**) collapse of the right flange.

**Figure 10:**
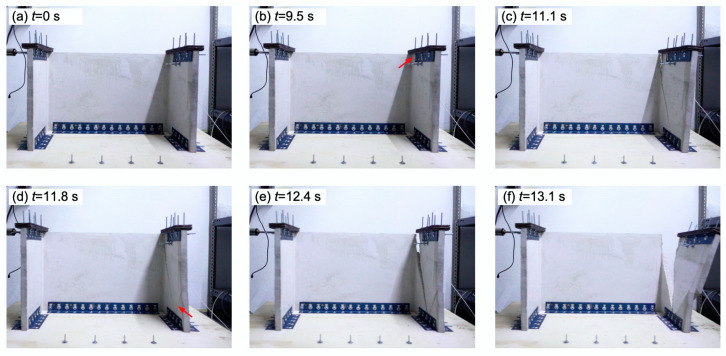
Collapse sequence of FRFC specimen #1: (**a**) start of the test; (**b**) formation of the first visible crack on the right flange (indicated by a red arrow); (**c**) development of the first crack; (**d**) formation of a secondary crack on the right flange (indicated by a red arrow); (**e**) development of the secondary crack; and (**f**) collapse of the right flange.

Figure 9 and Figure 10 show the collapse sequence captured by the camera of the first flange of the PFC specimen #1 and FRFC specimen #1, respectively. These two specimens had similar failure modes and for both specimens a main diagonal crack was observed on the right flange. Figure 9b–d shows the initiation of the first visible crack (indicated by a red arrow), the subsequent crack development and the collapse of the flange, respectively. For the PFC specimen #1, the time between the initiation of the first visible crack on the right flange and the collapse of that flange was 0.92 s. Figure 10b–f shows the initiation of the first visible crack, the subsequent crack development and the collapse of the flange, respectively. It can be seen in Figure 10d that a secondary diagonal crack developed on the right flange of the FRFC specimen #1. For this specimen, the time between the initiation of the first visible crack and the collapse of right flange was 3.59 s. As aforementioned, this longer time period between the initiation of the first visible crack and the collapse of the flange for the FRFC specimen is attributed to the enhanced integrity of the specimen, provided by the fibers. This enhancement of specimen integrity and mechanical properties of fiber-reinforced cement-based materials has been observed for different types of fiber-reinforcements, including natural [33,56], synthetic [32,57] and metallic fibers [58], and carbon nanotubes [59]. It has been reported that in fiber-reinforced concrete, the fibers prevent the concrete from cracks growing by forming connection bridges [60]. It is noted that in most cases, the video recording allowed for the identification of the moment when the first visible crack formed. It occurred when there was a change in the direction of motion of the shaking table, causing the maximum peaks of acceleration.

**Figure 11:**
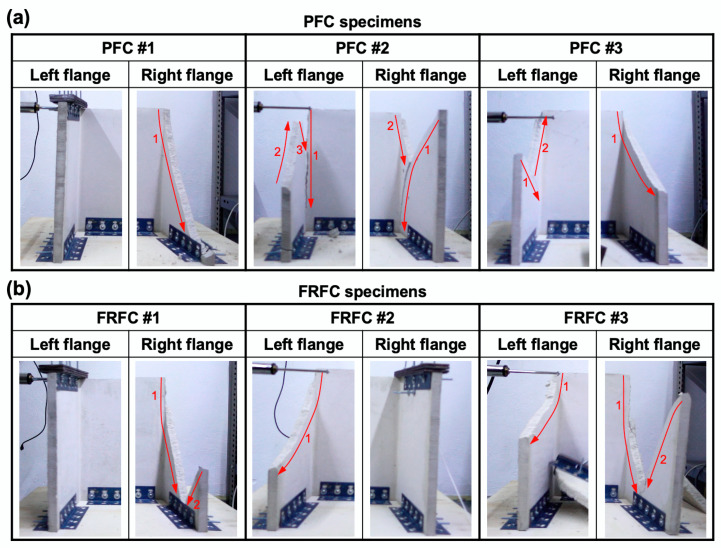
Failure modes observed during the shaking table tests of: (**a**) PFC specimens and (**b**) FRFC specimens.

Figure 11a,b shows the failure modes of the PFC and FRFC specimens, respectively. The red arrows indicate the direction of the crack growth. It can be seen in Figure 11 that the majority of the specimens exhibited one main diagonal crack that originated from the top corners of the wall. Some specimens exhibited a secondary crack on the same flange, and in one case, a third crack was also observed (Figure 11a, PFC #2, left flange).

### 3.2. Numerical Results

**Figure 12:**
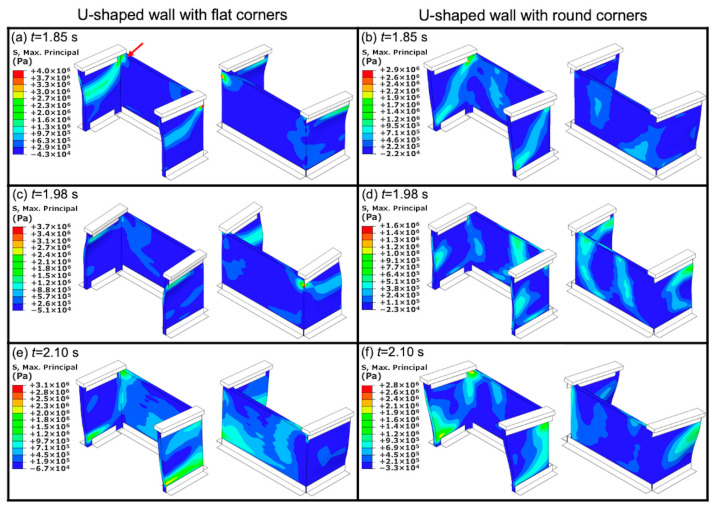
Contour plots of the maximum principal stress at different times of the shaking table test for U-shaped walls with flat corners and round corners: (**a**,**b**) *t* = 1.85 s; (**c**,**d**) *t* = 1.98 s; and (**e**,**f**) *t* = 2.10 s (for illustration purposes, the deformation in the direction of motion has been scaled up by a factor of 50 to visualize the bending of the specimens).

Figure 12 shows contour plots of the predicted maximum principal stress, for both U-shaped walls with flat corners and with round corners, at different times of the dynamic loading. The chosen times correspond to a full cycle (one wavelength) and show when the specimens were subjected to a change in direction, i.e., maximum acceleration. It is noted that in Figure 12, for illustration purposes, the deformation in the direction of motion has been scaled up by a factor of 50 to visualize the bending of the specimens [61]. It can be seen in Figure 12 that in most cases, the stress contour shows a diagonal band with high levels of stress at the flanges, and stress concentration at the top corners of the wall. These predicted observations are in agreement with the experimentally observed locations where the cracks formed (Figure 9 and Figure 10), and the diagonal cracks observed in the majority of the specimens (Figure 11). It can also be seen in Figure 12 that for the U-shaped wall with round corners, the levels of stress are lower than those observed in the U-shaped walls with flat corners. Figure 12 also shows that although there is stress concentration at the top corners of the U-shaped wall with round corners, the stress concentration is more evenly distributed throughout the specimen.

**Figure 13:**
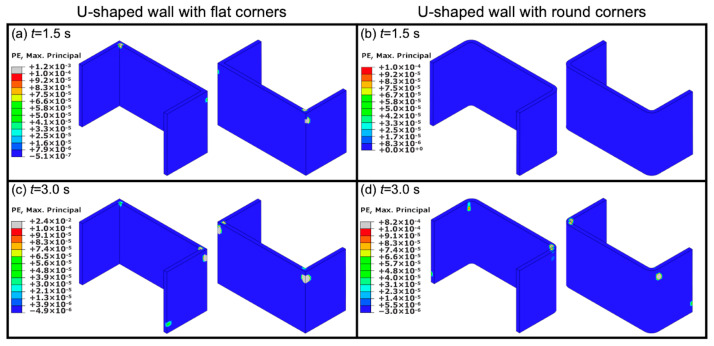
Contour plots of the maximum principal plastic strain at different times of the shaking table test for U-shaped walls with flat corners and round corners: (**a**,**b**) *t* = 1.5 s and (**c**,**d**) *t* = 3.0 s.

Figure 13 shows contour plots of the maximum principal plastic strain for both U-shaped walls with flat corners and with round corners at 1.5 and 3 s. It can be seen in Figure 13a that at *t* = 1.5 s, there is plastic strain at the top corners of the U-shaped wall with flat corners, while for the U-shaped wall with round corners, there is no plastic strain (Figure 13b). At *t* = 3.0 s, there is plastic strain in both types of U-shaped walls, however, the predicted value of the plastic strain for the U-shaped wall with round corners (0.0008) (Figure 13d) is much lower than that for the U-shaped wall with flat corners (0.024) (Figure 13c). These locations, in which there are large values of plastic strain, are more likely to develop cracks [62].

### 3.3. Discussion

The experimental results presented here have shown that henequen fiber-reinforcement can be used to improve the mechanical performance of U-shaped wall model specimens made of FRFC. This material has the potential to perform well in structural and seismic applications, and to be used for the fabrication of precast wall panels. However, we acknowledge that our results are preliminary and further experimental and numerical research has to be carried out to support the findings of this work. Further studies should include strategies to assess the effect of different fiber volume fractions and foamed concrete densities on the mechanical properties of the FRFC, an assessment of the durability of the henequen fibers in the cement-based foamed concrete, and further mechanical testing to fully understand the toughening mechanisms of the foamed concrete reinforced with natural fibers. For the U-shaped walls, it is necessary to test large scale models with different walling configurations, such as L-shaped walls. Dynamic testing should include different cyclic loadings and earthquakes excitations, and be performed using different loading directions. Although the experimental results presented here are limited, they provide useful knowledge about the behavior of fiber-reinforced materials and structures subjected to dynamic loading. These results could also be useful to validate numerical models that are used to predict seismic and dynamic behavior.

The numerical results showed that the design of the U-shaped wall is crucial to reduce stress concentration that may lead to the formation of plastic strain, cracking and subsequent failure. The FE simulations presented here are important from a design viewpoint because they show that material properties and geometrical characteristics can potentially be tailored to improve seismic performance. These FE simulations could be extended to predict the seismic response of FRFC walls with more complex geometries, including the simulation of a full-scale U-shaped wall or a complete house. However, more mechanical testing is needed to obtain a complete set of parameters for material model calibration and numerical simulation validation. Finally, robust material models, that include failure criterion, should be employed to obtain more accurate predictions.

## 4. Conclusions

In this work, the mechanical performance of U-shaped wall model specimens subjected to dynamic cyclic loading, by means of shaking table tests, was investigated. The U-shaped walls were made of foamed concrete without fiber-reinforcement (PFC) and with fiber-reinforcement (PFC). Both PFC and FRFC, with a target dry density of 900 kg/m^3^, were characterized using compression and tensile tests. These tests showed that the fiber-reinforcement improved the mechanical properties, toughness and ductility of the foamed concrete, which was attributed to the enhanced specimen integrity produced by the inclusion of the fibers. For the dynamic testing, the U-shaped walls were subjected to a 4-Hz sinusoidal base motion with an amplitude of 20 mm. The results showed that the U-shaped wall specimens made of FRFC performed better than those made of PFC because the time period prior to the collapse of the U-shaped walls was longer for the FRFC specimens, which was attributed to enhanced integrity of the specimens by the fibers. The finite element simulations proved to be a useful tool to improve the design of the U-shaped walls and predict stress concentration and the formation of plastic strain that may lead to cracking and failure during the dynamic cyclic loading. The results presented here showed that U-shaped walls made of FRFC have the potential to perform well in structural and seismic applications. However, these results are preliminary and further experimental and numerical research should be carried out to support the findings of this work.

## Figures and Tables

**Table 1:** Mechanical properties of PFC and FRFC.

Properties	PFC	FRFC
Density (kg/m^3^)	895.6 ± 4.1	930.3 ± 4.9
Compressive elastic modulus (MPa)	454.5 ± 36.5	461.2 ± 29.5
Compressive strength (MPa)	4.06 ± 0.25	4.16 ± 0.31
Compressive yield strain (mm/mm)	0.014 ± 0.001	0.015 ± 0.001
Tensile strength (MPa)	0.35 ± 0.02	0.48 ± 0.05
Tensile yield strain (mm/mm)	0.023 ± 0.010	0.027 ± 0.009

**Table 2:** Time period for the first visible crack and failure of the U-shape wall flanges.

U-Shaped Wall Specimen	Time of First Visible Crack on the First Flange (s)	Time of Collapse of the First Flange (s)	Time of First Visible Crack on the Second Flange (s)	Time of Collapse of the Second Flange (s)	Duration of the Test (s)
PFC #1	3.01 (RF) ^1^	3.93 (RF)	-	-	6.28 ^3^
PFC #2	2.58 (RF)	3.76 (RF)	3.80 (LF)	6.63 (LF)	9.96
PFC #3	6.21 (LF) ^2^	6.48 (LF)	6.92 (RF)	7.43 (RF)	9.35
FRFC #1	9.50 (RF)	13.09 (RF)	-	-	39.25
FRFC #2	41.57 (LF)	43.15 (LF)	-	-	51.16
FRFC #3	2.44 (RF)	6.45 (RF)	15.82 (LF)	16.62 (LF)	17.49

^1^ RF—Right Flange; ^2^ LF—Left Flange; ^3^ The data recording stopped prematurely.

## References

[1] Maccarini H., Vasconcelos G., Rodrigues H., Rodrigues H., Lourenço P.B. (2018). Out-of-plane behavior of stone masonry walls: Experimental and numerical analysis. Constr. Build. Mater..

[2] Park J., Towashiraporn P., Craig J.I., Goodno B.J. (2009). Seismic fragility analysis of low-rise unreinforced masonry structures. Eng. Struct..

[3] Bruneau M. (1994). State-of-the-art report on seismic performance of unreinforced masonry buildings. J. Struct. Eng..

[4] Ortega J., Vasconcelos G., Rodrigues H., Correia M., Lourenço P.B. (2017). Traditional earthquake resistant techniques for vernacular architecture and local seismic cultures: A literature review. J. Cult. Heritage.

[5] Poole D., Renique G. (2017). Cashing in on the quakes. NACLA Rep. Am..

[6] Godínez E., Tena A., Archundia H., Gómez A., Ruíz R., Escamilla J. (2019). Daños en viviendas localizadas en el sureste de méxico ocasionados por el sismo de tehuantepec del 7 de septiembre de 2017, mw = 8.2. Rev. Int. Ing. Estruct..

[7] Singh S.K., Ordaz M., Alcântara L., Shapiro N., Kostoglodov V., Pacheco J.F., Alcocer S., Gutiérrez C., Quaas R., Mikumo T. (2000). The oaxaca earthquake of 30 september 1999 (mw = 7.5): A normal-faulting event in the subducted cocos plate. Seism. Res. Lett..

[8] Banadaki H.M., Morshed R., Eslami A. (2019). In-plane cyclic performance of adobe walls retrofitted with near-surface-mounted steel rebars. Eng. Struct..

[9] Liu C., Nong X., Zhang F., Quan Z., Bai G.-L. (2019). Experimental study on the seismic performance of recycled concrete hollow block masonry walls. Appl. Sci..

[10] Betti M., Galano L., Vignoli A. (2015). Time-history seismic analysis of masonry buildings: A comparison between two non-linear modelling approaches. Buildings.

[11] Alshawa O., De Felice G., Mauro A., Sorrentino L. (2011). Out-of-plane seismic behaviour of rocking masonry walls. Earthq. Eng. Struct. Dyn..

[12] Saleem M.U., Numada M., Amin M.N., Meguro K. (2016). Shake table tests on frp retrofitted masonry building models. J. Compos. Constr..

[13] Chavez M., Meli R. (2011). Shaking table testing and numerical simulation of the seismic response of a typical Mexican colonial temple. Earthq. Eng. Struct. Dyn..

[14] Illampas R., Charmpis D.C., Ioannou I. (2014). Laboratory testing and finite element simulation of the structural response of an adobe masonry building under horizontal loading. Eng. Struct..

[15] Nezhad R.S., Kabir M.Z., Banazadeh M. (2016). Shaking table test of fibre reinforced masonry walls under out-of-plane loading. Constr. Build. Mater..

[16] Zhang S., Yang D., Sheng Y., Garrity S.W., Xu L. (2017). Numerical modelling of FRP-reinforced masonry walls under in-plane seismic loading. Constr. Build. Mater..

[17] Domínguez-Santos D., Ballesteros-Pérez P., Mora-Meliá D. (2017). Structural resistance of reinforced concrete buildings in areas of moderate seismicity and assessment of strategies for structural improvement. Buildings.

[18] Lemos J.V. (2019). Discrete element modeling of the seismic behavior of masonry construction. Buildings.

[19] Afrouz S.G., Razavi M.R., Pourkand A., Wilson C.M.D. (2019). Dynamic displacement of an aluminum frame using close range photogrammetry. Buildings.

[20] Borah B., Singhal V., Kaushik H.B. (2019). Sustainable housing using confined masonry buildings. SN Appl. Sci..

[21] Ferretti E., Pascale G. (2019). Combined strengthening techniques to improve the out-of-plane performance of masonry walls. Materials.

[22] Sciarretta F. (2020). Seismic retrofitting of traditional masonry with pultruded frp profiles. Appl. Sci..

[23] Corradi M., Castori G., Sisti R., Borri A., Pesce G.L. (2019). Repair of block masonry panels with cfrp sheets. Materials.

[24] Dong K., Sui Z.-A., Jiang J., Zhou X. (2019). Experimental study on seismic behavior of masonry walls strengthened by reinforced mortar cross strips. Sustainability.

[25] Domínguez-Santos D., Mora-Meliá D., Pincheira G., Ballesteros-Pérez P., Retamal-Bravo C. (2019). Mechanical properties and seismic performance of wood-concrete composite blocks for building construction. Materials.

[26] Tomaževič M., Gams M. (2011). Shaking table study and modelling of seismic behaviour of confined AAC masonry buildings. Bull. Earthq. Eng..

[27] Tomaževič M., Gams M. (2010). Seismic behaviour of confined autoclaved aerated concrete masonry buildings: A shaking table study. Mauerwerk.

[28] Dunn T.P., Van Zijl G., Van Rooyen A.S. (2018). Investigating a reinforced lightweight foamed concrete walling system for low-rise residential buildings in moderate seismic regions. J. Build. Eng..

[29] Ramamurthy K., Nambiar E.K., Ranjani G.I.S. (2009). A classification of studies on properties of foam concrete. Cem. Concr. Compos..

[30] Sayadi A., Tapia J.V., Neitzert T.R., Clifton G.C. (2016). Effects of expanded polystyrene (EPS) particles on fire resistance, thermal conductivity and compressive strength of foamed concrete. Constr. Build. Mater..

[31] Amran Y.M., Farzadnia N., Ali A.A. (2015). Properties and applications of foamed concrete; a review. Constr. Build. Mater..

[32] Falliano D., De Domenico D., Ricciardi G., Gugliandolo E. (2019). Compressive and flexural strength of fiber-reinforced foamed concrete: Effect of fiber content, curing conditions and dry density. Constr. Build. Mater..

[33] Mahzabin M.S., Hock L.J., Hossain M.S., Kang L.S. (2018). The influence of addition of treated kenaf fibre in the production and properties of fibre reinforced foamed composite. Constr. Build. Mater..

[34] Jones R., McCarthy A. (2005). Preliminary views on the potential of foamed concrete as a structural material. Mag. Concr. Res..

[35] Flores-Johnson E.A., Li Q.M. (2012). Structural behaviour of composite sandwich panels with plain and fibre-reinforced foamed concrete cores and corrugated steel faces. Compos. Struct..

[36] Islam M.S., Iwashita K. (2010). Earthquake resistance of adobe reinforced by low cost traditional materials. J. Nat. Disaster Sci..

[37] Ramezanianpour A., Esmaeili M., Ghahari S.A., Najafi M. (2013). Laboratory study on the effect of polypropylene fiber on durability, and physical and mechanical characteristic of concrete for application in sleepers. Constr. Build. Mater..

[38] Ramezanianpour A.A., Ghahari S.A., Khazaei A. Feasibility Study on Production and Sustainability of Poly Propylene Fiber Reinforced Concrete Ties Based On a Value Engineering Survey. Proceedings of the 3rd International Conference on Sustainable Construction Materials and Technologies (SCMT3).

[39] Roslan A.F., Awang H., Mydin A.O. (2012). Effects of various additives on drying shrinkage, compressive and flexural strength of lightweight foamed concrete (lfc). Adv. Mater. Res..

[40] Kearsley E., Visagie M. (2002). Properties of foamed concrete as influenced by air-void parameters. Concr. Beton.

[41] Hoyos C.G., Zuluaga R., Gañán P., Pique T.M., Vazquez A. (2019). Cellulose nanofibrils extracted from fique fibers as bio-based cement additive. J. Clean. Prod..

[42] Onuaguluchi O., Banthia N. (2016). Plant-based natural fibre reinforced cement composites: A review. Cem. Concr. Compos..

[43] Mydin A.O., Noordin N.M., Utaberta N., Yunos M.Y.M., Segeranazan S. (2016). Physical properties of foamed concrete incorporating coconut fibre. J. Teknol..

[44] Liu Y., Wang Z., Fan Z., Gu J. (2020). Study on properties of sisal fiber modified foamed concrete. IOP Conf. Ser. Mater. Sci. Eng..

[45] Flores-Johnson E.A., Yan Y.Z., Carrillo J.G., Gonzalez-Chi P.I., Herrera-Franco P.J., Li Q.M. (2018). Mechanical characterization of foamed concrete reinforced with natural fibre. Mater. Res. Proc..

[46] Valadez-Gonzalez A., Cervantes-Uc J., Olayo R., Franco P.J.H. (1999). Effect of fiber surface treatment on the fiber–matrix bond strength of natural fiber reinforced composites. Compos. Part B: Eng..

[47] Kozłowski M., Kadela M. (2018). Mechanical characterization of lightweight foamed concrete. Adv. Mater. Sci. Eng..

[48] Kadela M., Kozłowski M. (2016). Foamed concrete layer as sub-structure of industrial concrete floor. Procedia Eng..

[49] Zafra Pinacho D., Gastéllum Alvarado J.M. (2015). Catálogo de la vivienda vernácula en el estado de Oaxaca Caso: Distrito de Tlacolula. Estud. Sobre Conserv. Restaur. y Museol..

[50] De Leo A. (2015). Catálogo de Arquitectura Vernácula de Oaxaca.

[51] QUANSER (2019). Shake Table II Data Sheet, Quanser Inc., Markham, Canada. https://www.quanser.com/products/shake-table-ii/.

[52] Ali M., Briet R., Chouw N. (2013). Dynamic response of mortar-free interlocking structures. Constr. Build. Mater..

[53] ABAQUS (2015). Abaqus Analysis User’s Guide.

[54] Chiacchiarelli L.M., Cerrutti P., Flores-Johnson E.A. (2019). Compressive behavior of rigid polyurethane foams nanostructured with bacterial nanocellulose at low and intermediate strain rates. J. Appl. Polym. Sci..

[55] Amarnath Y., Ramachandrudu C. Properties of Foamed Concrete with Sisal Fibre. Proceedings of the 9th International Concrete Conference 2016: Environment, Efficiency and Economic Challenges for Concrete, University of Dundee.

[56] Ahmad W., Farooq S.H., Usman M., Khan M., Ahmad A., Aslam F., Al Yousef R., Alabduljabbar H., Sufian M. (2020). Effect of coconut fiber length and content on properties of high strength concrete. Materials.

[57] Szeląg M., Szeląg M. (2019). Evaluation of cracking patterns of cement paste containing polypropylene fibers. Compos. Struct..

[58] Setti F., Ezziane K., Setti B. (2020). Investigation of mechanical characteristics and specimen size effect of steel fibers reinforced concrete. J. Adhes. Sci. Technol..

[59] Carriço A., Bogas J.A., Hawreen A., Guedes M. (2018). Durability of multi-walled carbon nanotube reinforced concrete. Constr. Build. Mater..

[60] Kakooei S., Akil H.M., Jamshidi M., Rouhi J. (2012). The effects of polypropylene fibers on the properties of reinforced concrete structures. Constr. Build. Mater..

[61] Vazquez-Rodriguez J., Flores-Johnson E.A., Herrera-Franco P.J., Gonzalez-Chi P.I. (2017). Photoelastic and numerical analyses of the stress distribution around a fiber in a pull-out test for a thermoplastic fiber/epoxy resin composite. Polym. Compos..

[62] Colombo I.G., Colombo M., Di Prisco M., Pouyaei F. (2018). Analytical and numerical prediction of the bending behaviour of textile reinforced concrete sandwich beams. J. Build. Eng..

